# Supplementary material for: Antibacterial, Mutagenic Properties and Chemical Characterisation of Sugar Bush (Protea caffra Meisn.): A South African Native Shrub Species
Source: Plants (Basel). 2020 Oct 9;9(10):1331. doi: 10.3390/plants9101331 (PMC7600625; doi:10.3390/plants9101331)
Supplement: Supplementary file 1 [file plants-09-01331-s001.pdf]

Supplementary Materials: The following are available online at www.mdpi.com/xxx/s1, Table S1 and Figures S1-3.

Table S1. Minimum inhibitory concentration values (MIC, mg/mL) of aqueous extracts of *Protea caffra* screened against drug-sensitive and -resistant bacterial strains.

	Minimum inhibitory concentration (MIC, mg/mL)				
**Plant part**	***Ec***	***Ef***	***Kp***	***Sa***	***Sa* D**
Bark	2.5	2.5	2.5	2.5	2.5
Flowers	2.5	2.5	2.5	2.5	2.5
Leaves	2.5	2.5	2.5	2.5	2.5
Seeds	2.5	2.5	2.5	2.5	2.5
Twigs	2.5	2.5	2.5	2.5	2.5
**Neomycin (µg/mL)**	0.78	0.39	1.6	0.65	6.25

Ec = *Escherichia coli*; *Ef* = *Enterococcus faecalis*; *Kp* = *Klebsiella pneumoniae*; *Sa* = *Staphylococcus aureus*, *Sa* D = Penicillin -resistant *S. aureus*.

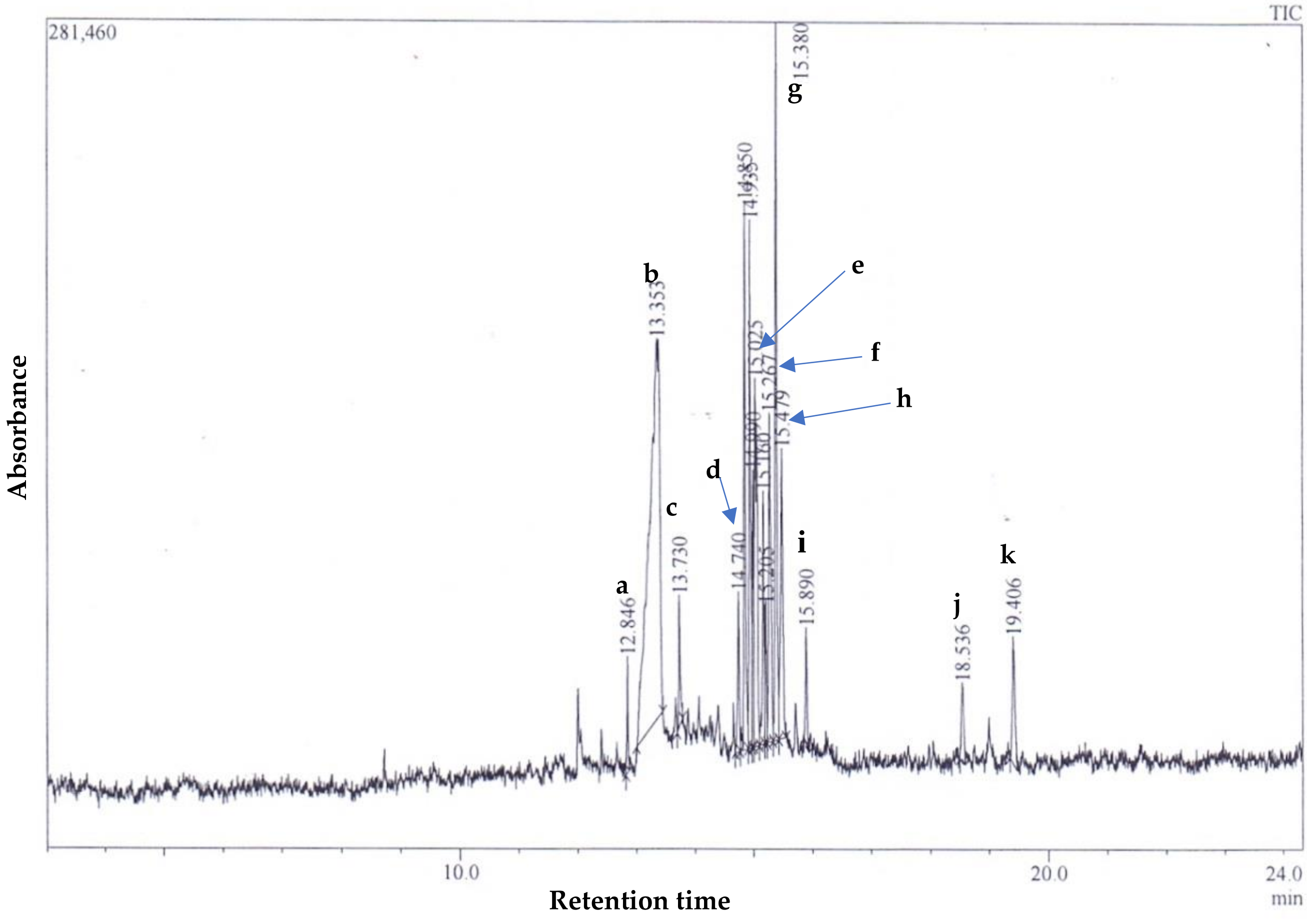

Figure 1. GC-MS chromatogram of a cold ethyl acetate sub-fraction obtained from methanolic extracts of *Protea caffra* twigs. a = Phenol 2,4-bis(1,1-dimethylethyl); b = Polygalitol; c = 1,3-Benzenediol, 4-propyl-; d = 1,2-Bis(p-acetoxyphenyl) ethanedione; e = 1-Adamantanecarboxylic acid, 2-propenyl; f = Phenol, 2-methyl-4-(1,1,3,3-tetramethylbutyl)-; g = Phenol, 4-(1,1,3,3-tetramethylbutyl)-; h = Phenol, 2-methyl-4-(1,1,3,3-tetramethylbutyl)-; i = 1-Nonadecanol; j = Phthalic acid, butyl tridecyl ester; k = 1-Heptacosanol.

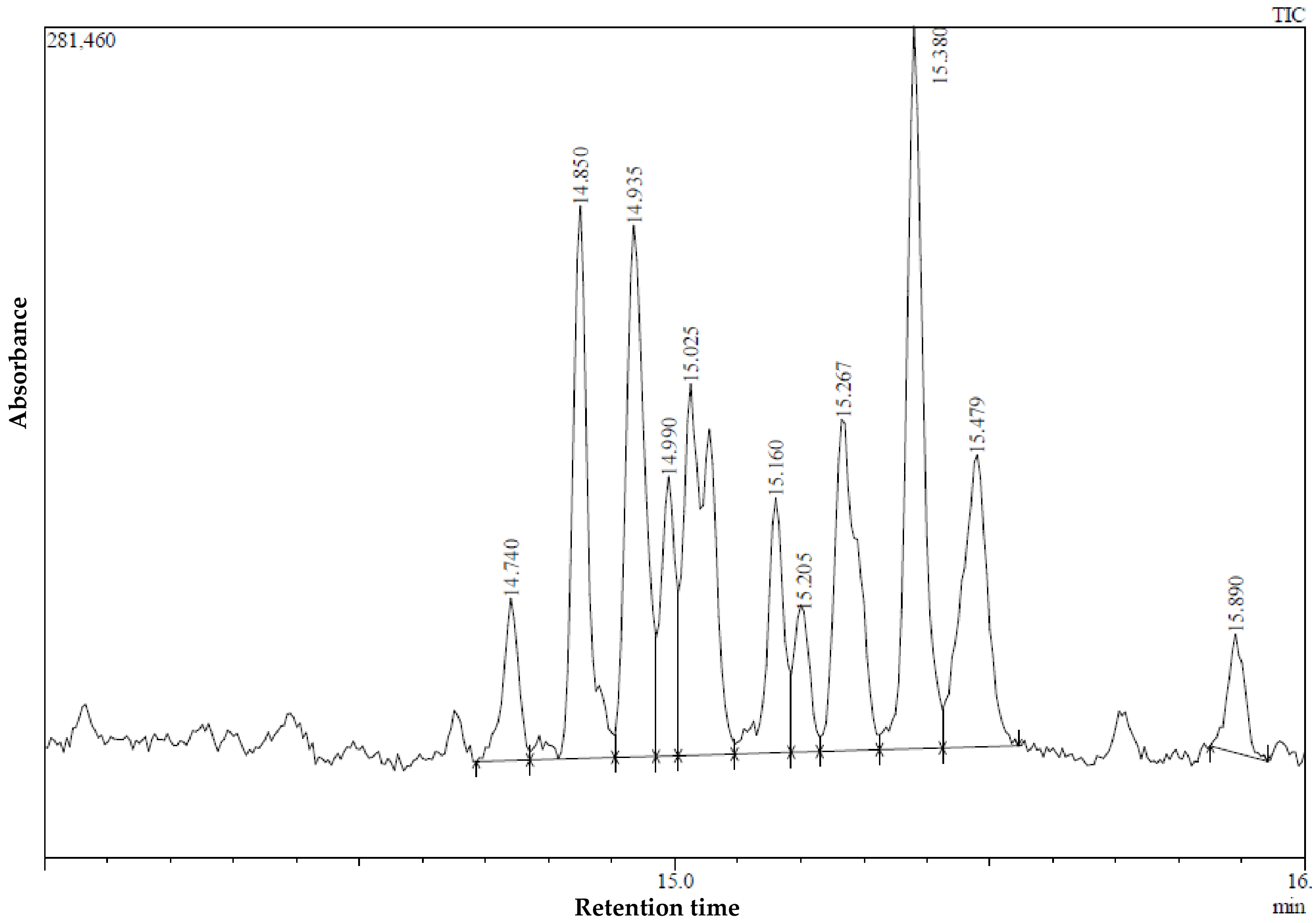

Figure S1.1: Extension of a GC-MS chromatogram (14-16 mins) of a cold ethyl acetate sub-fraction obtained from methanolic extracts of *Protea caffra* twigs.

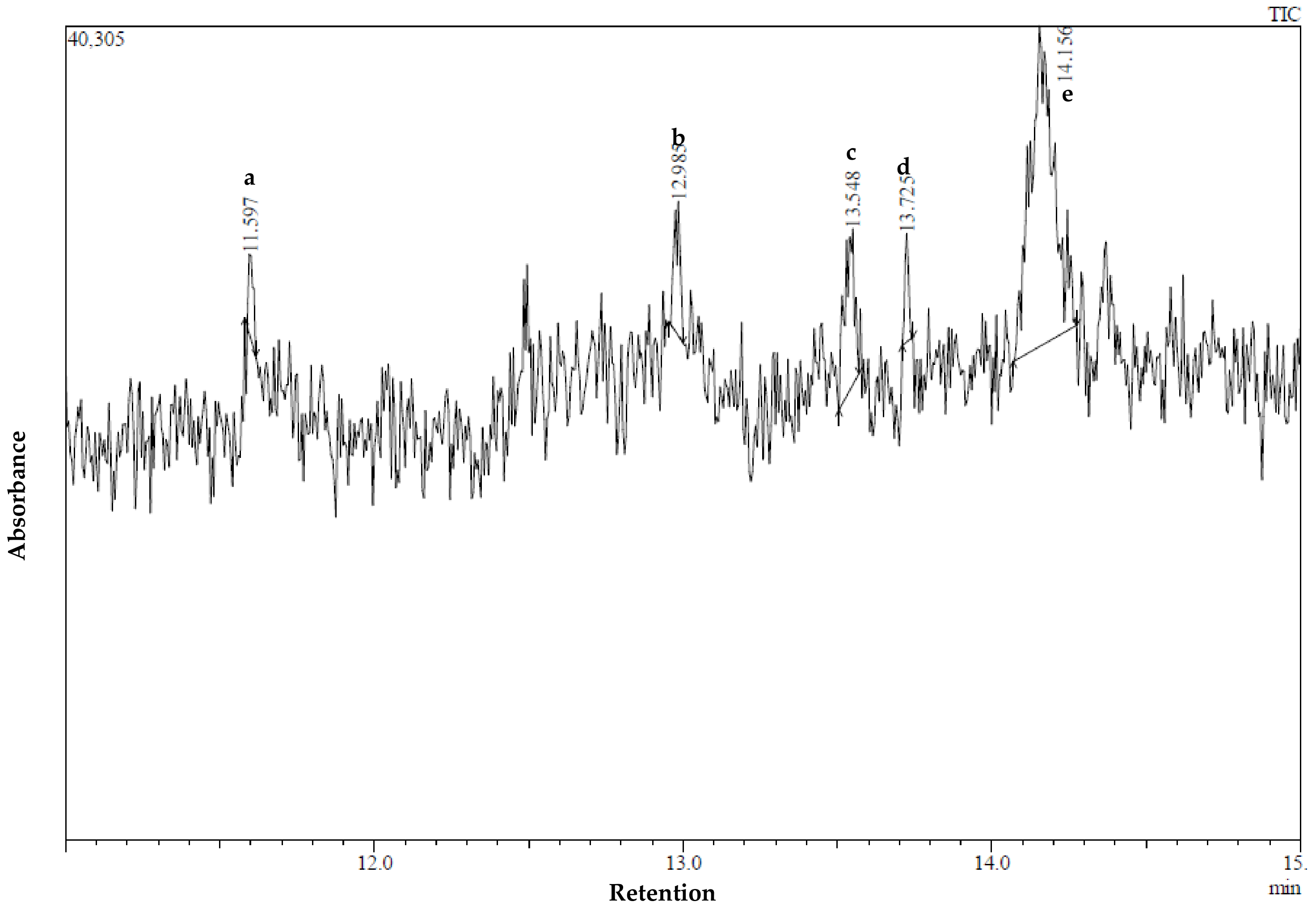

Figure S2: GC-MS chromatogram of a hot ethyl acetate sub-fraction obtained from methanol extracts of *Protea caffra* twigs. a = ß-Glucopyranose, 1,6-anhydro-, 1,6-anhydro-; b = Polygalitol; c = 1,3,5-Benzenediol, 4-propyl-; d = 1,3-Benzenediol, 4-propyl-; e = 1-Heptacosanol.

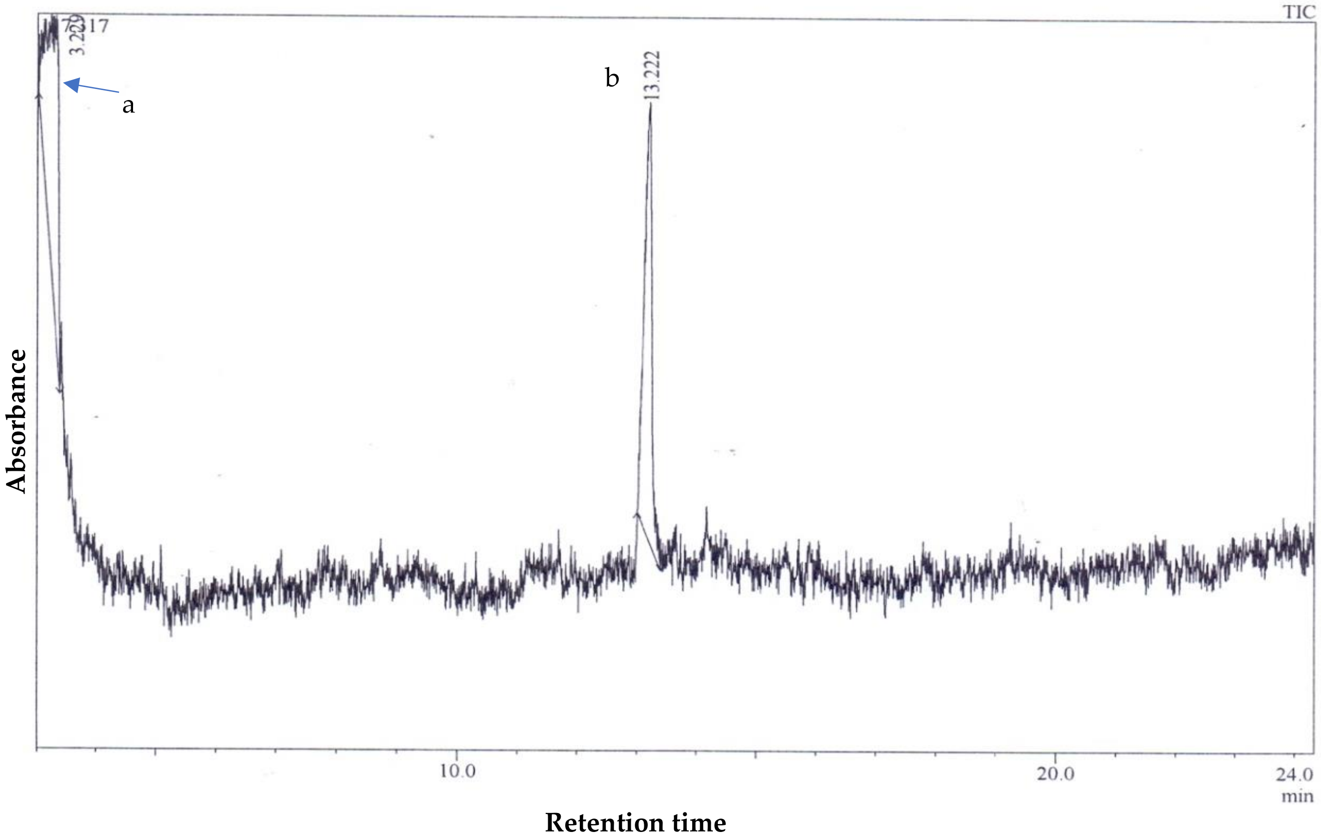

Figure S3: GC-MS chromatogram of an acetone sub-fraction obtained from methanolic extracts of *Protea caffra* twigs. a = Oxalyl acid; b = Polygalitol.
